# Preparation of Pure and Stable Chitosan Nanofibers by Electrospinning in the Presence of Poly(ethylene oxide)

**DOI:** 10.3390/ijms17111790

**Published:** 2016-10-26

**Authors:** Solomon Mengistu Lemma, Frédéric Bossard, Marguerite Rinaudo

**Affiliations:** 1 Laboratoire Rhéologie et Procédés (LRP), University of Grenoble Alpes-CNRS, 38000 Grenoble, France; solomonmengistu@gmail.com (S.M.L.); frederic.bossard@ujf-grenoble.fr (F.B.); 2 Biomaterials Applications, 6 Rue Lesdiguières, 38000 Grenoble, France

**Keywords:** electrospinning, chitosan, PEO, nanofiber, stability

## Abstract

Electrospinning was employed to obtain chitosan nanofibers from blends of chitosans (CS) and poly(ethylene oxide) (PEO). Blends of chitosan (*M*_W_ (weight-average molecular weight) = 102 kg/mol) and PEO (M (molecular weight) = 1000 kg/mol) were selected to optimize the electrospinning process parameters. The PEO powder was solubilized into chitosan solution at different weight ratios in 0.5 M acetic acid. The physicochemical changes of the nanofibers were determined by scanning electron microscopy (SEM), swelling capacity, and nuclear magnetic resonance (NMR) spectroscopy. For stabilization, the produced nanofibers were neutralized with K_2_CO_3_ in water or 70% ethanol/30% water as solvent. Subsequently, repeated washings with pure water were performed to extract PEO, potassium acetate and carbonate salts formed in the course of chitosan nanofiber purification. The increase of PEO content in the blend from 20 to 40 w% exhibited bead-free fibers with average diameters 85 ± 19 and 147 ± 28 nm, respectively. Their NMR analysis proved that PEO and the salts were nearly completely removed from the nanostructure of chitosan, demonstrating that the adopted strategy is successful for producing pure chitosan nanofibers. In addition, the nanofibers obtained after neutralization in ethanol-aqueous solution has better structural stability, at least for six months in aqueous solutions (phosphate buffer (PBS) or water).

## 1. Introduction

Researchers have been working on the processing of chitosan to produce new biomaterials, especially developed for biomedical applications [1,2,3,4,5,6,7,8,9,10]. The advantage of this natural polymer is that it becomes water soluble in acidic conditions due to –NH_2_ protonation as soon as its degree of acetylation is lower than 0.5. Then, processing of chitosan is relatively easy and it can be used as a fiber, nanofiber, film, capsule, bead, sponge, gel, powder or tablet, based on its insolubility in neutral medium (i.e., when chitosan is in the –NH_2_ form). Chitosan is often difficult to characterize but its main characteristics are its weight-average molecular weight (*M*_W_) and the average degree of acetylation (DA) as discussed previously [11]. It is an interesting biodegradable and biocompatible polymer with antibacterial and antifungal properties often described in the literature [12]. In addition, chitosan is stabilized by H-bond network in solid state, providing good mechanical properties under film or fiber materials. Biomedical applications are the main applications proposed for chitosan: in drug delivery, as a gene delivery vehicle, for encapsulation of sensitive drugs, in medical textiles, in guided bone regeneration, as a scaffold for nerve tissue regeneration, and in wound healing. The nanofibers are also used under membranes for fine filtration, and for adsorption of toxic metals [13]. The advantage of the nanofibrous filter media is their high surface area per unit of mass and strong mechanical strength [14]. A wide range of methods has been used to produce chitosan-based nanofibrous materials, typically electrospinning which has received a lot of attention in several scientific studies [1,15]. Electrospinning of chitosan has been improved, optimizing the affecting factors such as the solution properties (viscosity, surface tension and conductivity) [16,17,18], processing parameters and ambient conditions [16,19,20,21,22].

Chitosan has already been electrospun using different acid-based solvents [23,24]. For instance, acetic acid used at concentration from 90% down to 1% (or 0.17 M) gives good fibers [16,25]. It is important to mention that pure chitosan fibers have rarely been obtained in acetic acid at 90% and 70% [19], but mainly with trifluoroacetic acid (TFA) [14,26] or mixture of TFA and dichloromethane [23,27]. The use of ionic liquid was also mentioned with 1,1,1,3,3,3-hexafluoro-2-propanol [28]. More generally, it has been shown that fine nanofibers can be obtained in chitosan blends with poly (vinyl alcohol) (PVA) [23,25,29,30], gelatin or collagen [2,7,31,32,33,34], silk fibroin [5], polycaprolactone [35] but mainly with poly(ethylene oxide) (PEO) [6,17,18,36,37,38,39,40,41]. Usually, to generate composite fibers the spinning solutions are obtained by mixing the two polymers solutions prepared separately in the same solvent. The processing of chitosan blends occurs by mono or coextrusion [20], and core-shell structured PEO-chitosan nanofibers are also obtained, leading to a hollow nanofiber by removing PEO after washing with water [42]. Interesting, shape-memory behavior was reported with relatively high PEO content [40]. Good spinnability of PEO is recognized, and interaction with chitosan favors the processing [17] in addition to its low toxicity [43].

The chitosan molecular weight from which nanofibers have been obtained varies from 85 kg/mol [42] up to 570 kg/mol [18,27,39], nevertheless the real molecular weight is often difficult to obtain precisely due to the presence of aggregates [11]. Most generally moderate molecular weights in the range of 100 to 200 kg/mol, used in different solvents. The concentration used for electrospinning is directly related to the chitosan molecular weight which imposes the solution viscosity. The concentration adopted is in the range of 20 to 50 g/L in different solvents [19,23,24,29,38]. It has been demonstrated that low *M*_W_ (as 30 kg/mol) or higher *M*_W_ (as 398 kg/mol) are not able to give good fibers in 90% acetic acid [24].

Due to swelling of the chitosan blend, crosslinking was proposed using glutaraldehyde [25,26] or genipin [44]. Nevertheless, there are no very promising results after those chemical reactions. Therefore, another more direct way should be modification of the intrinsic properties of chitosan, which becomes insoluble in the –NH_2_ form. Then, due to the H-bond network established between chitosan chains, no additional chemical reaction is needed. To obtain stabilization of the chitosan nanofibers in aqueous medium, especially for biomedical applications, it is necessary to prevent solubilization of chitosan due to its net charge under its salt form. The stability is usually tested by the degree of swelling in phospahe buffer (PBS) or study of the morphology of fibers by scanning electron microscopy (SEM). This problem is not often discussed in the literature. The use of acid with a low boiling temperature (such as acetic acid) allows extraction of excess acid and water constituting the solvent left after the electrospinning in ambient conditions. Then, a neutralization step is important to obtain the insoluble chitosan. For this purpose, it was proposed that the nanofiber membrane be immersed in 5 M NaOH or 5 M Na_2_CO_3_ aqueous solutions for 3 h at ambient conditions [27] or in 1 M K_2_CO_3_ for 3 h at 25 °C [14].

With respect to the applications of these chitosan materials obtained by electrospinning, it must be pointed out that they are important for cell cultures [5,37], tissue engineering [6], and also for ion adsorption [14,39,41] or water treatment [15]. It is also necessary to mention that not only electrospun chitin and chitosan are produced but also chitin derivatives [24,45,46].

In our work, the electrospinning of chitosan-based nanofibers was developed in order to obtain a higher yield of chitosan. For that purpose, two different techniques were used. Firstly, solutions of PEO and solutions of chitosan were mixed to produce nanofibers. Secondly, powdered PEO was added into chitosan solutions. Due to toxicity of the solvents able to be used, the acetic acid in absence of any additive was chosen at the concentration of 0.5 M corresponding to maximum of chitosan solubility [47]. The development of chitosan nanofibers without toxic solvent allows for production of a better candidate for biomedical, food and pharmaceutical applications. The change of the molecular weight and weight ratio of PEO in the chitosan blend were investigated and related with the electrospun nanofiber thickness. The primary aim was achieved to develop stable and pure chitosan nanofibers without PEO and remaining salts. Furthermore, the influence of neutralization step on the morphology stability of these fibers was also covered.

## 2. Results and Discussions

Production of regular electrospun nanofibers from pure chitosan solutions is known to be challenging. Due to this, chitosan/PEO (CS/PEO) blends were used to enhance the spinnability of different *M*_W_ chitosans in a common solvent. The influence of PEO was examined in the range of high amount of chitosan compared with PEO with the objective of producing pure chitosan fibers.

### 2.1. Chitosan/PEO (CS/PEO) Compatibility Based on Solution Viscosity

First of all, the solvent adopted was fixed at 0.5 M acetic acid after testing 90% acetic acid which is too volatile. Electrospinning of pure chitosan solution leads to the formation of beads instead of fine nanofibers. Then, taking into account data from literature [10,17,18,35,36,42], it was decided to mix chitosan with PEO, allowing for better electrospinning.

**Figure 1:**
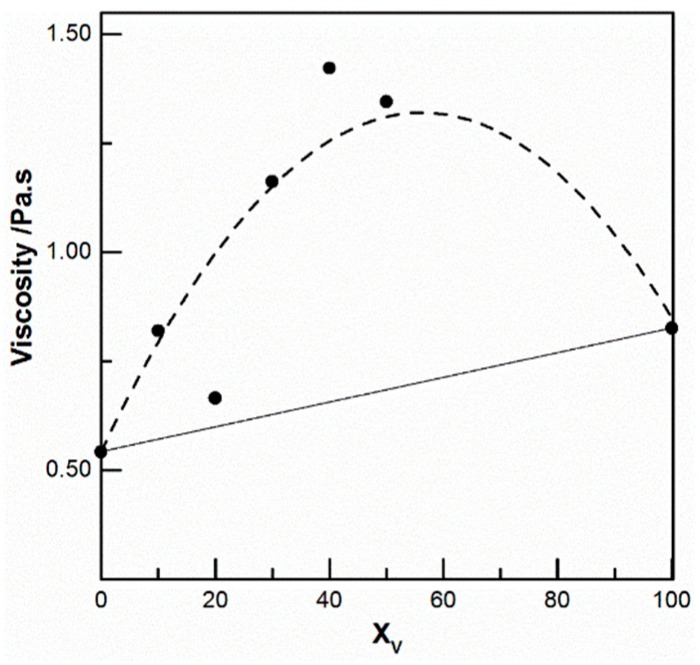
Variation of the viscosity of chitosan CS-102/ PEO (M = 1000 kg/mol) mixtures in 0.5 M acetic acid at 0.5 s^−1^ as a function of weight fraction of PEO (*X*_v_). Total polymer concentration 5% *w*/*v*. PEO: poly(ethylene oxide); CS: Chitosan.

In parallel, to test the compatibility of these two polymers, the viscosities of mixed solutions prepared at 5% (*w*/*v*) were determined. All the solutions tested were Newtonian at low shear rate. The main data are given in Figure 1 where the viscosities at 0.5 s^−1^ were plotted as a function of CS/PEO ratio. The total polymer concentration remains constant and equal to 5% *w*/*v*. These results confirm the synergistic interaction between the two polymers in the chosen solvent used as mentioned in the literature [17,48,49]. An optimum viscosity value was obtained around a 50/50 (*v*/*v*) ratio but there was no significant change in the viscoelastic behavior of the solutions.

**Figure 2:**
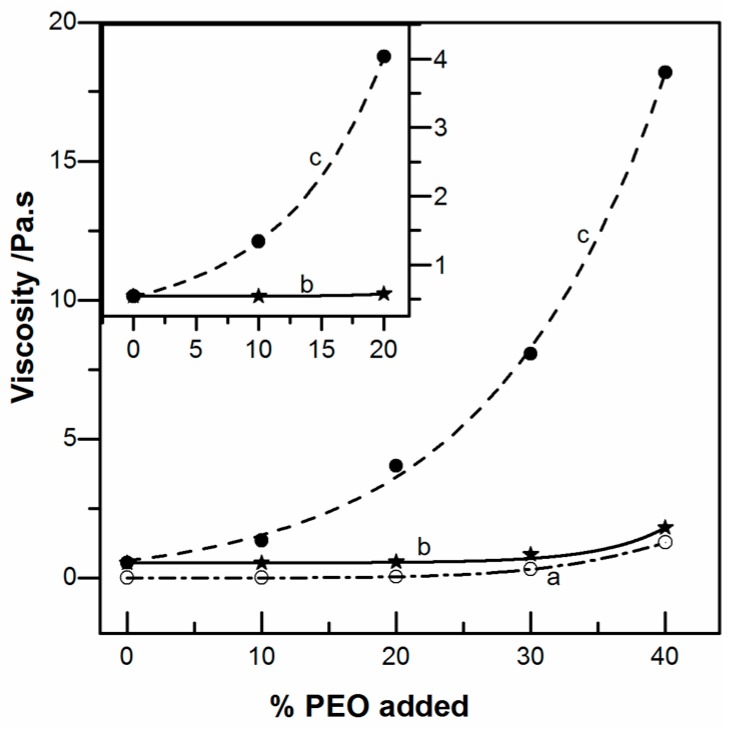
Variation of the viscosity at 0.5 s^−1^ of added PEO (M = 1000 kg/mol) expressed in percent of total polymer concentration in the mixtures. (a) PEO solution in 0.5 M acetic acid ೦; (b) additivity calculated for each values of “a” added into pure chitosan CS-102 ⋆ and (c) blends chitosan CS-102/PEO as a function of the weight fraction of PEO ●.

A second technique used to prepare the chitosan/PEO solution consisted of adding solid PEO into 5% (*w*/*v*) chitosan solution. As shown in Figure 2, the viscosity increased progressively, depending on the concentration and molar mass of PEO used. In these conditions, the chitosan concentration remained constant (5% *w*/*v*). Up to the ratio 60/40, G’ < G’’ in the entire range of frequency was tested but the solutions become non-Newtonian. These data confirm the cooperative interaction between chitosan and PEO especially when the experimental data from Figure 2, curve c is compared to the additivity calculated in Figure 2, curve b. All these solutions were electrospun without any difficulty, except pure chitosan and chitosan blended with 10% PEO.

### 2.2. Polymers Concentration and Molecular Weight Influences

A number of experiments were carried out to determine the optimum processing conditions and blend ratio of CS/PEO in order to get nanofibrous matrix. First, PEO (300 kg/mol) at 5% (*w*/*w*) solution in 0.5 M acetic acid was electrospun to optimize the production of fibers as shown in the Table 1. The microscope observation confirms that PEO can give fibers at this molecular weight with adopted electrospinning parameters. In the same processing conditions, chitosan (102 kg/mol) solution at 5% (*w*/*w*) in 0.5 M acetic acid was also tested to form fibers. Unlike PEO, electrospinning of this chitosan solution generated mainly beads (Table 1).

Therefore, blending of the two polymers solutions at a total fixed polymer concentration (5% *w*/*w*) was selected as an alternative option to obtain composite fibers. In addition, the pump rate was also kept at 50 µL/h in all electrospun samples. Exceptionally, blended CS/PEO (90/10) and CS/PEO (70/30) formed elongated beads and fibers at 30 and 20 µL/h pump rate, respectively. However, Table 1 shows that regardless of the blend ratio and change in the corresponding processing parameters, well-structured nanofibers were not obtained. One of the reasons might be that mixing the two solutions causes a decrease in the final chitosan and PEO concentrations.

Thus, electrospinning of blend CS/PEO at 5% *w*/*w* from chitosan (102 kg/mol) and PEO (300 kg/mol) solutions in acetic acid was not successful in producing bead free fibers. For this reason, powdered PEO (with five different molecular weights) were added to 5% (*w*/*w*) chitosan (102 kg/mol) solutions to control CS/PEO ratios. The addition of powdered PEO helps to avoid the dilution effect of the final chitosan concentration and allows producing nanofibers as reported in Table 2. The processing parameters such as pump rate, collection distance and applied voltage were kept the same as those adopted for data given in Table 1. Nevertheless, in some cases, the collection distance and pump rate were changed to optimize the processing parameters. Microscope images of these different blended samples showed that the addition of powdered PEO favors the formation of fibers in contrary to those obtained in the conditions adopted in Table 1. In general, the addition of powdered PEO into chitosan solutions to produce electrospun chitosan-PEO fiber matrix has never before been reported.

Table 2 firstly allowed the role of PEO molecular weight at a CS/PEO ratio 80/20 to be shown. At lower PEO molecular weights no fibers were obtained. On the hand, fibers were obtained with chitosan (102 kg/mol) blended with PEO, with molecular weights equal to or higher than M 1000 kg/mol. This M PEO was selected for further production of chitosan-PEO matrices (shown in Table 3). In addition, it is observed that, when the M of PEO is larger (M 8000 kg/mol), fibers were obtained at an even lower PEO content (at CS/PEO ratio 90/10). From complementary results shown in Table 2, the influence of PEO (M 300 kg/mol) content was demonstrated: bead free fibers were obtained for CS/PEO ratios lower than 80/20 in relation with the good spinnability of PEO. Finally, the influence of the chitosan molecular weight was examined. Results obtained when powdered PEO (M 1000 kg/mol) was added in chitosan solutions (CS-102 at 5% and CS-500 at 1.4%) are given in Table 3.

Different composition ratios were covered as shown in Table 3. Overall, the results confirm that the blend with chitosan (102 kg/mol) gave better fibers compared to the higher molecular weight chitosan (500 kg/mol) at the same CS/PEO ratio. Particularly, using the higher *M*_W_ chitosan, it was impossible to increase the concentration above 1.4% *w*/*w*. This is due to the increased viscosity of the blend when PEO dissolved in this chitosan solution. These results indicate that chitosan with moderate *M*_W_ favors fiber production, allowing an increase chitosan content. Similar conclusions on chitosan *M*_W_ influence were obtained previously for chitosan dissolved in TFA-DCM system and in 90% acetic acid respectively [9,24].

Taking into account our experimental data, 5% (*w*/*v*) chitosan solutions (102 kg/mol)/powdered PEO (1000 kg/mol) were selected for preparing the chitosan/PEO ratios 90/10, 80/20, 70/30 and 60/40 (*w*/*w*). These blended solutions were used to produce electrospun nanofibers in optimal conditions and to perform further physicochemical analyses.

### 2.3. Morphology and Stability of the Chitosan Nanofibers

#### 2.3.1. Electrospun Chitosan/PEO Nanofibers

**Figure 3:**
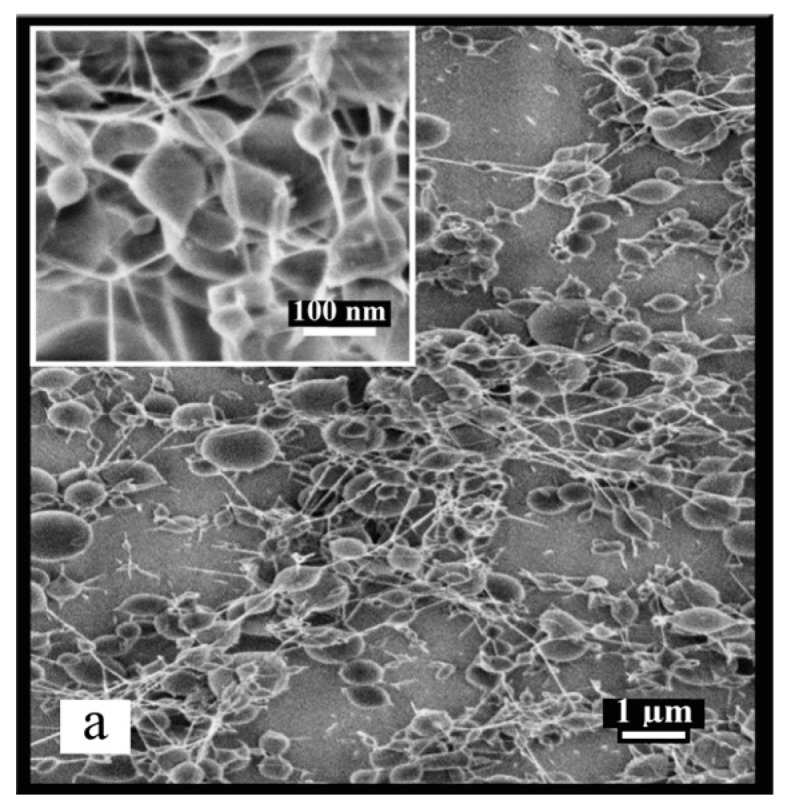

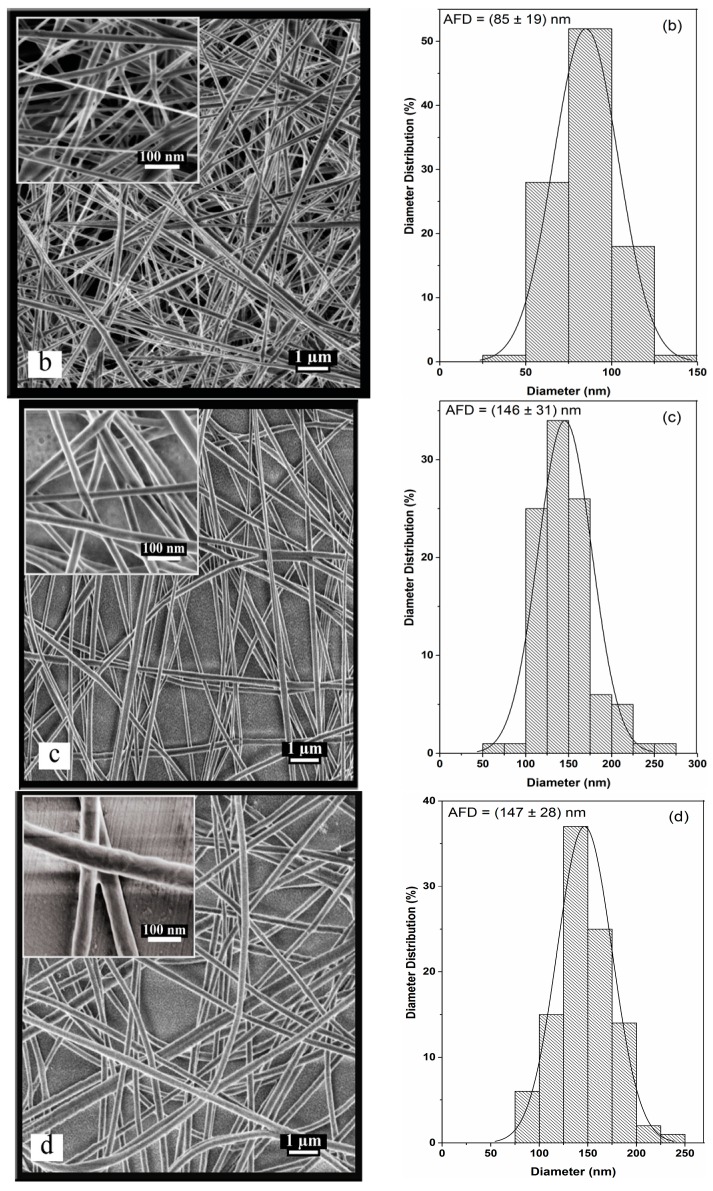
Representative SEM images and fiber diameter distributions of the electrospun nanofibers obtained from blend of powdered PEO into chitosan solutions (CS/PEO) at weight ratio (**a**) 90/10; (**b**) 80/20; (**c**) 70/30; and (**d**) 60/40. The insets show the high magnification images. The fiber diameter distributions of each are shown on the right. AFD: average fiber diameter; SEM: scanning electron microscopy.

A selected chitosan 5% (*w*/*w*) solution/powdered PEO, with molecular weight of 102 and 1000 kg/mol, respectively, were used to obtain stable nanofibrous chitosan as shown in Figure 3. The processing parameters of electrospinning were optimized at pump rate 20 µL/h, applied voltage of 20 kV and tip to collector distance of 13 cm. Furthermore, the conductivity of the blends decreased with respect to the reduction of weight ratio of protonated chitosan in CS/PEO solutions. With respect to the increment of 10% powdered PEO in the blends of CS/PEO from 10% to 40%, these solutions have conductivity values of 7.21 ± 0.01, 6.95 ± 0.02, 6.53 ± 0.01 and 6.22 ± 0.03 mS/cm, respectively. This decrement of the conductivity leads to an increase in the average fiber diameter (AFD) due to lower stretching of the solution at constant strong electric field [17]. 

As can be seen in Figure 3a, the results of the blended CS/PEO (90/10) exhibit more nano-beads with average diameters of (244 ± 61) nm. These beads are connected with fibers having AFD of 46 ± 13 nm. This result led to the assumption that the beads are formed from chitosan and the connecting fibers from PEO. It is evident that 10% of PEO in the blend did not prevent chitosan from forming bead-free fibers and stable Taylor cone. This result is consistent with results in other studies using different solvents [9,17,37]. Accordingly, powdered PEO content increased to 20% in the blend, favoring the optimized processing parameters and producing nano-scale fibers of chitosan. At 80/20 ratio of CS/PEO, well-structured fine nanofibers were formed with average fiber diameter (AFD) of 85 ± 19 nm, together with very few elongated beads (Figure 3b). In the case of 70/30 and 60/40, the smooth nanofibers free of beads were obtained; determined AFD was 146 ± 31 nm in Figure 3c and 147 ± 28 nm in Figure 3d, respectively. These results clearly revealed that bead free nanofibers can be produced when the content of chitosan prepared at 5% initial concentration is equal to or less than 80% in the blend. In addition, the AFD of the blend at 70/30 is very close to 60/40. However, the increased content of powdered PEO from 20% to 30% in the blends increased the AFD by 41%. This indicates that the increased content of chitosan together with optimal blending ratio of powdered PEO has significant effect on nanofiber size.

#### 2.3.2. Stabilization of Chitosan Nanofibers

**Figure 4:**
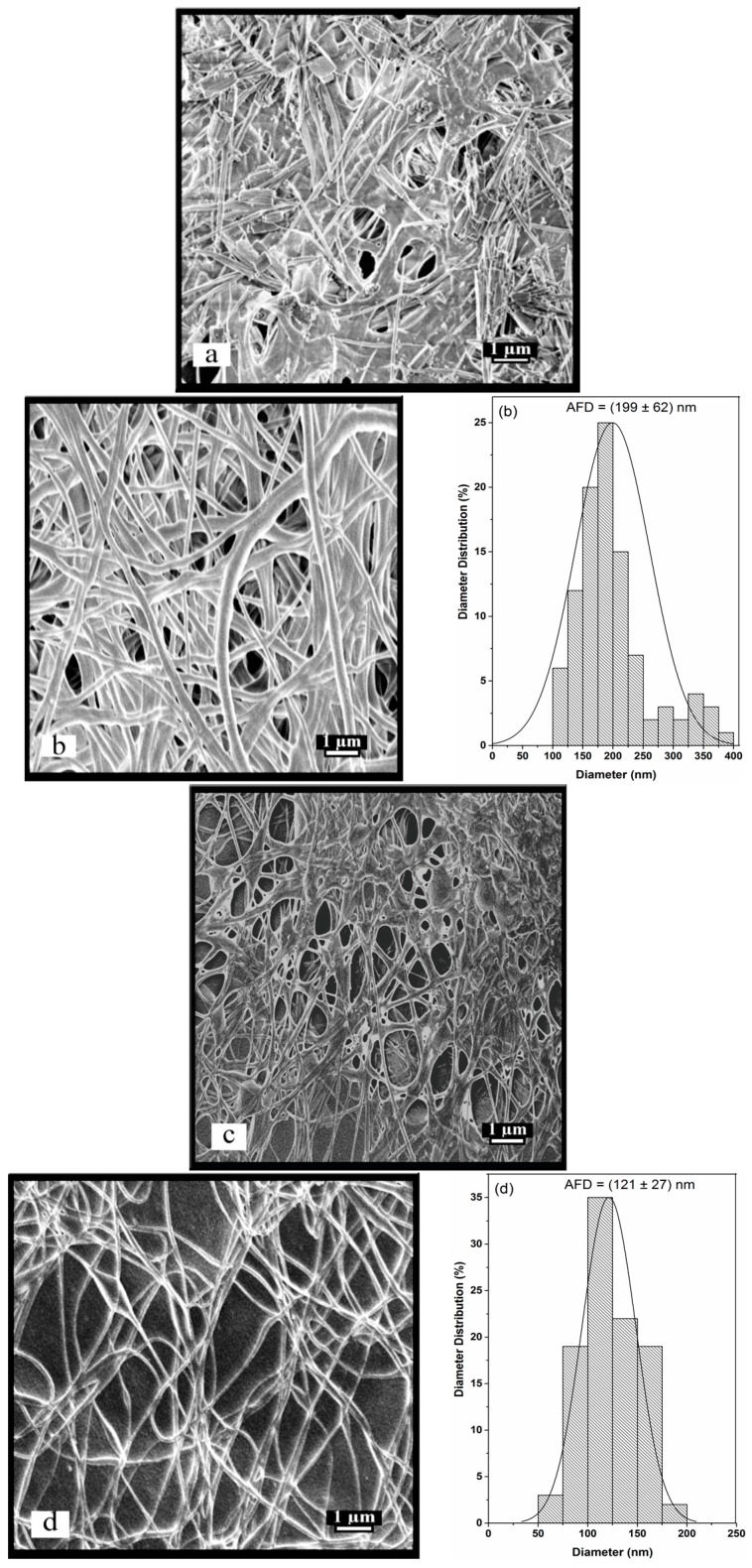
The SEM images of the membrane surface of chitosan/PEO at different ratios. (**a**) 60/40 neutralized in water; (**b**) 60/40 neutralized in ethanol/water mixture; (**c**) 80/20 neutralized in water; and (**d**) 80/20 neutralized in ethanol/water mixture.

Electrospinning of CS/PEO at ratio of 80/20 and 60/40 nanofibers were chosen to examine structural stability after repeated extraction of PEO. Specifically, the nanofibers from CS/PEO (80/20) were studied in reason of their high content of chitosan in the blend. The lower content of chitosan at the ratio CS/PEO (60/40) was investigated also and compared with CS/PEO (80/20). To obtain stable chitosan nanofibrous membranes, washing out PEO from CS/PEO nanofibers is necessary. However, before total PEO extraction, neutralization of the chitosan fibers needs to be performed. For that purpose and following the literature, the nanofibers membranes were immersed in aqueous 1 M K_2_CO_3_ for two days with exchange of the washing solution four times at 25 °C [14,24]. This allowed for neutralizing of the chitosan nanofiber membranes, becoming insoluble in aqueous medium under the –NH_2_ form. In addition, it is known that many charged polysaccharides are insoluble in ethanol/water mixtures. For instance, it has been shown that in presence of 2% acetic acid, chitosan is insoluble at ethanol yield over 40% [50]. Then, a new post-treatment was proposed, involving the neutralization of chitosan in presence of ethanol, a non-solvent of chitosan. Stabilization was performed in 70/30 ethanol/aqueous 1 M K_2_CO_3_ treatment to get the insoluble -NH_2_ form chitosan, followed by water washing. Then, the two methods of neutralization were compared using K_2_CO_3_ in 100% water or 70%/30% ethanol-water mixture. These treatments were carried out to check the influence of neutralization conditions on the stability of the chitosan nanofibers structure as depicted in Figure 4.

Figure 4 shows the SEM images of CS/PEO before and after immersion in both neutralizing media. The nanofibrous membranes neutralized in water failed to preserve the chitosan nanofiber structure due to partial dissolution when the initial fibers under the acetate ionic form were immersed in 1 M K_2_CO_3_. This is evidenced by the existence of a film-like structure (Figure 4a,c). Typically, in the SEM image, CS/PEO (60/40) lost its nano-structured fibers (Figure 4a) more than CS/PEO (80/20) as shown in Figure 4c. This may be due to larger amount of PEO washed out from the composite nanofibrous structure and partial swelling of protonated chitosan at first contact with aqueous solution. On the contrary, SEM images analyses confirmed the stability of nanofibrous structures after treatment with ethanol-water (70/30) as shown in Figure 4b,d. Generally, the AFD determined after immersion in ethanol-water (70/30) was increased by around 30% for CS/PEO (80/20) and 33% for CS/PEO (60/40) compared with the initial size (Figure 3b,d). This size increment may be due to the swelling of the fibers. This is in good agreement with previous studies which indicated that washing of chitosan membranes increased the size of the nanofibers by swelling without change of fibrous morphology [37,51]. In this case, it is shown that the fiber morphology is perfectly preserved and consists of pure chitosan as demonstrated by nuclear magnetic resonance (NMR). To conclude, chitosan neutralization in ethanol-water mixture is suitable for preserving the nanofiber structure as shown in Figure 4b,d. A similar conclusion was obtained previously when 3 M NaOH in aqueous solution of methanol was used [51].

### 2.4. Characterization of Chitosan Nanofibers

The electrospun nanofibers obtained are composed of interacting PEO and chitosan in the acetate salt form which are water soluble in acidic conditions. For this purpose, excess of acetic acid and water may be easily separated after processing by evaporation in ambient conditions. When these fibers were immersed in PBS buffer at pH = 7.4 or in water, the fibers were swollen and PEO was progressively solubilized, modifying their properties. In our approach, to obtain pure chitosan fibers, the electrospun fibers were immersed in K_2_CO_3_ to neutralize chitosan and then in water to extract PEO, and potassium acetate and carbonate salt formed. The proposed neutralization post-treatment was applied in presence of ethanol to get pure chitosan nanofibers with preserved morphology (Figure 4b,d). After repeated washing in water, the residual final fibers should be composed of pure chitosan in the –NH_2_ form. The analysis of fibers and role of different steps of purification were tested by NMR spectroscopy. Examples are given in the following section.

#### 2.4.1. NMR Analysis and Composition of the Fibers

**Figure 5:**
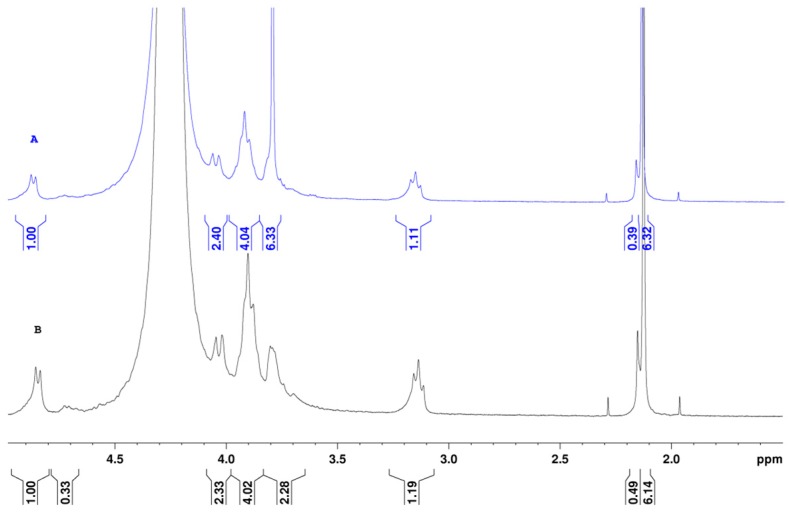
^1^H-NMR spectrum for the CS-102/PEO (80/20) mixture solubilized in 0.5 M acetic acid with added D_2_O (**A**) and CS-102 solubilized in 0.5 M acetic acid with added D_2_O (**B**).

Nuclear magnetic resonance (NMR) spectroscopy was used to investigate chemical changes of nanofibers of CS/PEO. This helps to identify the remaining PEO and acetic acid in the chitosan nanofibers. In Figure 5, the NMR spectrum of a CS/PEO (80/20) blend as used for electrospinning (Figure 5A) was compared with that of chitosan in the same solvent (Figure 5B). The integrals of the main signals are given in the Figure. Considering the chitosan spectrum (Figure 5B), the following signals are identified at 2.15 ppm corresponding to the –CH_3_ protons and at 3.13 ppm for the proton in –C_2_ position. The ratio between these two integrals allows to determine the DA of chitosan [52].

With respect to the analysis of the mixture of CS/PEO solution prepared in acetic acid and added D_2_O (Figure 5A), the signal of –CH_2_– from PEO at 3.73 ppm is superimposed with the signal of one chitosan proton. Therefore, the quantitative determination of PEO content should be overestimated. Nevertheless, this thin signal allows for a conclusion on the presence or absence of PEO in the fibers analyzed after purification. In the two spectra, a large signal at 2.13 ppm (with two satellites) allows us to determine the acetic acid concentration used for electrospinning when the exact chitosan concentration is known.

**Figure 6:**
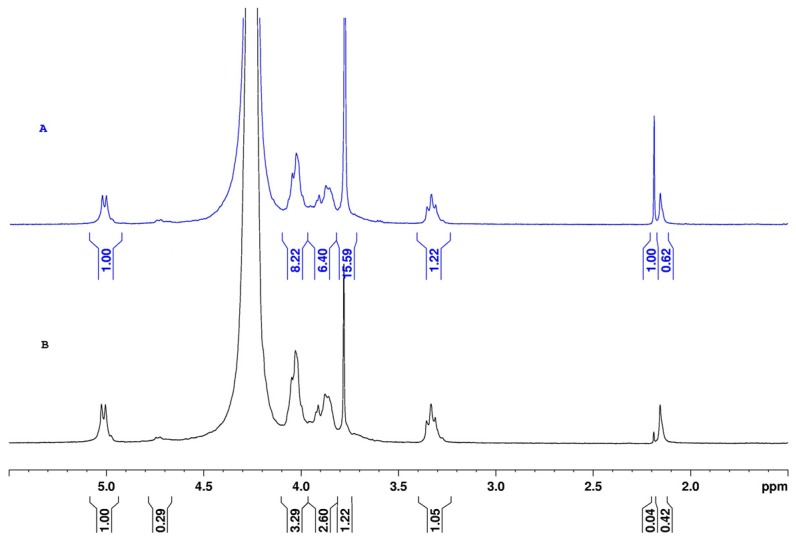
As spun fibers from the mixture CS/PEO (80/20) dissolved in acidic D_2_O (**A**); Stabilized and washed chitosan fibers from the mixture CS-102/PEO 80/20 dissolved in acidic D_2_O (**B**).

After electrospinning, the fibers were maintained at ambient conditions for one week; excess of acetic acid and water were evaporated to stabilize the material. Then, the composition was investigated after dissolution in D_2_O in acid conditions (DCl) (Figure 6). Firstly, a chemical shift was noted for all the signals compared with those obtained in acetic acid (+0.2 ppm in DCl) and inversion in the respective chemical shifts of the –CH_3_ of acetyl groups (at 2.15 ppm) and residual acetate salt from acetic acid (at 2.18 ppm).

From spectrum A, the amount of acetate groups was estimated and corresponded to a large part of the chitosan $-{\mathrm{NH}}_{3}^{+}$ counterions formed in presence of acetic acid. The proton of acetyl substituents also gave the DA, which was not modified in the electrospinning and washing processes. The PEO content in the material was clearly determined at 3.86 ppm but overestimated due to the overlap with one chitosan proton. After treatment with K_2_CO_3_ and water, the NMR spectrum was simplified as shown in Figure 6B. The signal for the acetate –CH_3_ groups was nearly suppressed: the ratio between the –CH_3_ signals of acetate and acetyl equaled 1.60 in the initial fibers (A) and became lower than 0.1 (B). In addition, only a small amount of PEO remained: the ratio of proton integral of –CH_2_– from PEO and that of chitosan H-2 equaled 1.22/1.05 which is negligible in comparison of the same ratio in Figure 6A giving 15.59/1.22.

^1^H-NMR is an interesting technique for analyzing the composition of the fibers regardless of the processing conditions. It is demonstrated that the treatment proposed allows obtaining nearly pure chitosan nanofibers in the example discussed in this work.

#### 2.4.2. Composition and Degree of Swelling of Chitosan Nanofibers

Comparison of the dried weight before and after the post-treatments allowed determination of the amount of PEO extracted from the nanofibers. These data were confirmed by the NMR results. In general, the post-treatment proposed in this work allowed us to obtain pure chitosan nanofibers which were perfectly stable after neutralization in ethanol-aqueous medium at pH > 6.5. In these conditions at neutral pH (immersion into salt, buffer and water), the cohesion of membrane was retained and they were easy to manipulate. Then, the degree of swelling was determined with respect to the purified never-dried chitosan fibers. An example is given for the chitosan fibers obtained with the ratio 80/20. The degree of swelling based on pure chitosan nanofibers was equal to 5.0 ± 0.2 g/g in water. In PBS buffer, the degree of swelling was equal to 4.0 ± 0.2 g/g in relation with ionic concentration of the buffer. In the same conditions, after K_2_CO_3_ in ethanol-water treatment and water extraction, the degree of swelling of fibers produced from 60/40 mixture was 6.7 ± 0.1 g/g, probably due to larger accessibility of the never dried fibers after the extraction of 40% PEO. In addition, the dried weight was lower than the initial one, corresponding to the chitosan left as insoluble under the –NH_2_ form after extraction of water soluble PEO as mentioned previously. In fact, it is very important to determine the degree of swelling on the basis of the final dried weight of pure chitosan.

After drying, the fibers were rehydrated in pure water to determine the water regaining capacity. The results give small decrease of the original swelling, with values of 4.0 ± 0.2 and 2.42 ± 0.2 g/g for CS/PEO (80/20) and CS/PEO (60/40), respectively.

## 3. Materials and Methods

### 3.1. Materials

Two different chitosan (CS) samples were used in this work. The first sample of chitosan was used as a moderate molecular weight (*M*_W_) and it is identified as CS-102. Its commercial name is Seacure 143 with *M*_W_ = 102 kg/mol and degree of acetylation (DA) = 0.12. This was obtained from Pronova Biopolymer (now Novamatrix, Sandvika, Norway). The second sample had a higher molecular weight; it is identified as CS-500. Its name is Kitomer and it is produced by Marinard Lte, Montreal, QC, Canada) with *M*_W_ = 500 kg/mol and DA = 0.19. PEO with different molecular weights M (8 × 10^3^, 5 × 10^3^, 1 × 10^3^, 3 × 10^2^, 1 × 10^2^ kg/mol), acetic acid (≥99.7 %), ethanol and K_2_CO_3_ were purchased from Sigma-Aldrich (Munich, Germany). Deionized water (from Egis Eau, Grenoble, France) was used as solvent to make up the solutions. PBS buffer was provided by Gibco Life Technologies (osmolality 280–315 mOsm/kg; ref 10010023, Gaithersburg, MD, USA) for study of chitosan membrane stability. All reagents and polymers were used as received without further purification.

### 3.2. Sample Preparation

Chitosan (CS-102 and CS-500) solutions were prepared separately at 5% (*w*/*v*) and 1.4% (*w*/*v*) in 0.5 M acetic acid, respectively. These solutions were prepared at room temperature under magnetic stirring (100 rpm) for four days to obtain homogeneous solutions. In the same manner, poly(ethylene oxide) with different molecular weights were solubilized at 5% *w*/*v* in 0.5 M acetic acid under magnetic stirring. Each chitosan (CS) samples were mixed with the solution of PEO with weight ratio (in %) of 90/10, 80/20 70/30 and 60/40. Similarly, the same concentrations of CS-102 and CS-500 were blended with powdered PEO. Then, chitosan concentration remained equal to 5% and 1.4% (*w*/*v*), respectively with weight ratios of chitosan to PEO of 90/10, 80/20 70/30 and 60/40. The weight ratios were expressed as weight of chitosan or PEO by total polymer weight. All the solutions were kept under constant shaking at 100 rpm for one day at room temperature to achieve complete mixing. This addition of powder (PEO) in the solution of chitosan was selected to avoid the dilution of the final chitosan in the polymer blend solution when mixing the two polymer solutions. These solutions were kept at rest more than 30 min prior to electrospinning in order to remove air bubbles at room temperature. Ionic conductivities of the blended CS/PEO solutions were measured using a conductivity meter (CRISON CM 35, Barcelona, Spain) at room temperature.

### 3.3. Electrospinning

The prepared solutions were placed in a 5 mL plastic syringe fitted with a 21-gauge stainless steel needle. The syringe pump delivered solutions at specified flow rate vertically (model: KDS Legato 200, KD Scientific, Holliston, MA, USA), and electrospun with an applied voltage of 20 kV between the electrodes using a homemade dual high voltage power supplier (±30 kV, iseq GMBH, Radeberg, Germany). Then, the nanofibers were deposited on a rectangular grounded aluminum foil collector kept at either 10, 11, 13 or 15 cm away from at the tip of the needle. The experiments were carried out at room temperature in closed Plexiglas^®^ box with relative humidity ranging between 50% and 60%. The produced nanofibers matrices were left in ambient conditions to evaporate excess of acetic acid and water prior to further analyses.

### 3.4. Characterization of Nanofibers

#### 3.4.1. Morphology of the Nanofibers Membranes

The SEM analyses of the samples were performed at CMTC-INP, Grenoble, France. The morphology of electrospun nanofibers membranes, including the washed samples were observed with a scanning electron microscope (Zeiss ultra 55 SEM FEG, Oberkochen, Germany) operated at 1 kV. The nanofibers samples were coated with 1 nm gold/palladium layer for 10 s prior to SEM imaging. The average fiber and bead diameter (AFD) was calculated by randomly selected diameter of 100 nanofibers/nano-beads from each sample.

#### 3.4.2. Determination of Swelling Capacity

Weighted initial nanofiber membrane cut in pieces were immersed in 1 M K_2_CO_3_ solution in water or ethanol/water (70/30) mixture to neutralize the chitosan. Further, nanofibers membranes were washed four times daily for three days with deionized water until neutral pH was achieved in order to obtain removal of the salt formed from chitosan solutions (potassium acetate), K_2_CO_3_ excess and PEO. The following step was stabilization in water or PBS buffer (pH = 7.4) for 24 h. Lastly, the membranes were dried at room temperature for further determination of the swelling capacity. This dried weight decreases compared to the initial dried weight indicating PEO extraction as confirmed by NMR experiments. 

The swelling of the nanofibrous membranes was examined in terms of water loss between swollen state in water at neutral pH and final dried weight at room temperature. The wet swollen samples were weighed after blotting with tissue paper to remove excess surface water (W_w_). Accordingly, the dried samples were also weighted repeatedly until the mass became constant (W_d_). The measurements were carried out three times each. The average data were taken for the determination of swelling ratio S using the following equation:

$$S\;\left(g/g\right)=\left(\frac{W_{w}-W_{d}}{W_{d}}\right)$$

where W_w_ (g) is the weight of the swollen nanofibrous mat and W_d_ (g) is the weight of the samples after drying at room temperature.

Rehydration of dried samples was tested using the same conditions after two days in water at room temperature.

#### 3.4.3. Stabilization of Chitosan Nanofibers

The membrane stabilization was controlled by neutralization and regeneration of chitosan under the –NH_2_ form, which is insoluble in water over pH = 6.5. For this purpose, two different approaches were followed. The produced nanofibers from the blend of PEO (powder) added into CS-102 solution at 5% (*w*/*v*) were washed in aqueous or ethanol-aqueous mixture at (70/30) in the presence of 1 M K_2_CO_3_. After these first treatments, the membranes were repeatedly washed to remove water soluble PEO and salt excess. At the same time, the morphological change exhibited by extraction of PEO and swelling of the matrices was controlled for each sample. The effect of morphology and composition of the fibers changes were studied after drying at room temperature for 24 h by SEM and NMR, respectively. In addition, prolonged stability up to six months of CS/PEO nanofiber structure was investigated after initial immersion and neutralization of the membranes in 70/30 ethanol-aqueous solutions. In addition, the extraction of PEO was confirmed by difference between the dried weight of the initial membrane and the dried weight left after K_2_CO_3_, water or buffer post-treatments. The resulting stable material would then be pure chitosan nanofibers.

#### 3.4.4. NMR Characterization of Nanofibers

The composition of the nanofibers in chitosan, PEO and acetic acid (or acetate) remaining in the fibers were determined by ^1^H-NMR at 80 °C on a Bruker Avance III 400 spectrometer (Bruker, Billerica, MA, USA). Selected samples were used to analyze the NMR spectrum and exemplify the change in the nanofibers compositions. Nanofiber samples of 7 to 10 mg were dissolved in 1 mL D_2_O in presence of a stoichiometric amount of DCl. In a different test, 0.2 mL of chitosan solution and of CS/PEO mixed solution (80/20) in 0.5 M acetic acid were added to 1 mL D_2_O. Analysis of spectra allows determination of the amount of acetic acid remaining in the dried samples and yield of PEO remaining in the samples after washing in different conditions.

#### 3.4.5. Rheological Behavior

Rheological characteristics were studied using a ARG2 rheometer from TA Instruments (New Castle, DE, USA) with cone-plate geometry; the cone has a diameter of 50 mm, a 2°angle and a 60-μm gap. The temperature is controlled at 20 °C by a Peltier plate. Steady-state flow experiments were performed in the range of 0.05 to 10 s^−1^. The dynamic oscillation measurements were carried out for frequency sweeps between 0.5 to 10 Hz with 1% strain, corresponding to the linear viscoelastic region (LVR).

## 4. Conclusions

In this study, production and morphology of chitosan nanofibers were investigated using different molecular weights of chitosan, PEO and the blend of different CS/PEO ratios in 0.5 M acetic acid solution. The electrospinning process parameters were optimized to achieve stable chitosan nanofibers from the blends of CS/PEO. Particularly, the blend of chitosan with molecular weight of 102 kg/mol and PEO of 1000 kg/mol were used to produce nanofibers for further exploring the structural and chemical changes. The results have demonstrated that CS/PEO solutions containing 20 wt % and more than 20 wt % of powdered PEO solubilized into the chitosan 5% (*w*/*w*) solution improved spinnability, producing bead-free nanofibers. It was found that decreasing chitosan content in CS/PEO ratio from 80/20 to 60/40 leads to an increase in the nanofiber diameters from 85 to 147 nm, respectively. Image analyses on the nanofiber matrix from blends of CS/PEO (60/40) and (80/20) demonstrate that, after neutralization in 70% of ethanol aqueous solution, chitosan fiber morphology is stable in the aqueous solution at pH > 6.5. Particularly, the obtained nanofibers membranes preserved morphological structure after repeated water washings while the opposite was observed by neutralization in water at room temperature. In addition, the swelling capacity of these produced nanofibers of CS/PEO (80/20) and (60/40) matrix was around 5 and 6.7 (g/g), respectively. Degree of rehydration was slightly lower than these swelling values of never dried fibers. This suggests that chitosan nanofibers structures can be a good biomaterial candidate for biomedical, food, pharmaceutical and other applications due the use of toxic free solvents. In addition, the NMR analysis revealed that a complete extraction of PEO, acetic acid, potassium acetate and carbonate contents from nanofibers matrix was attained. In general, we successfully prepared pure stable electrospun chitosan nanofibers without using strong and/or toxic solvents. This optimized process can be used for further production of blend of CS/PEO nanofibers, particularly for stable chitosan nanofibers in wide range of applications.

## Figures and Tables

**Table 1:** Electrospun pure chitosan, PEO (300 kg/mol) and blends of CS/PEO at total polymer concentration (50 mg/mL) at 20 kV applied voltage, 50 µL/h pump rate and 15 cm collection.

CS (mg/mL)	PEO (mg/mL)	CS *M*_W_ (kg/mol)	CS/PEO (*w*/*w*)%	^β^ Electrospun Product
0	50	0	0/100	Fibers
50	0	102	0/100	Beads ^a^
45	5	102	90/10	Beads
40	10	102	80/20	Fibers + beads
35	15	102	70/30	Fibers + beads ^b^
30	20	102	60/40	Fibers + beads
25	25	102	50/50	Fibers + beads

^β^ Beads and fibers examined using Leica DM LM microscope (50×); ^a^ elongated bead obtained at 30 µL/h and ^b^ fibers having beads along them obtained at 20 µL/h.

**Table 2:** Composition of electrospun solutions obtained with different molecular weights PEO added into chitosan CS (*M*_W_ = 102 kg/mol) in 0.5 M acetic acid solution at 20 kV applied voltage.

CS (mg/mL)	PEO (mg/mL)	PEO M (kg/mol)	CS/PEO (*w*/*w*)%	Pump Rate (µL/h)	^β^ Electrospun Products
50	12.0	100	80/20	20, 30, 50 & 80	Beads ^a^
50	12.0	300	80/20	50	Beads
50	12.0	1000	80/20	50 & 100	Fibers
50	12.0	5000	80/20	20	Fibers ^b^
50	12.0	8000	80/20	80	Fibers
40	5.6	8000	90/10	50	Fibers
40	5.6	5000	90/10	10	Fibers + beads
50	10.0	300	80/20	20	Fibers + beads ^c^
50	20.0	300	70/30	20	Fibers
50	30.0	300	60/40	50	Fibers
50	40.0	300	55/45	50	Fibers

^β^ Beads and fibers examined using Leica DMLM microscopes (50×). In all cases the collection distance is 15 cm, except “a” used both 15 & 10 cm, b = 10 cm and c = 11 cm.

**Table 3:** Electrospun blends of chitosans having different molecular weights with PEO (1000 kg/mol).

CS (mg/mL)	PEO (mg/mL)	CS-*M*_W_ (kg/mol)	CS/PEO (*w*/*w*)%	Pump Rate (µL/h)	^β^ Electrospun Products
35.8	4.2	102	90/10	20	Beads
32.0	8.0	102	80/20	50	Fibers + beads
28.6	11.4	102	70/30	50 ^a^	Fibers
13.5	1.6	500 *	90/10	50 ^c^	Fibers + beads
12.8	3.4	500 *	80/20	50	Fibers + beads
12.3	4.9	500 *	70/30	50	Fibers + beads
11.2	7.8	500 *	60/40	20 ^b^	Beads

* 1.4% chitosan solution. ^β^ Beads and fibers examined using Leica DMLM microscopes (50×). The collection distance was 20 cm except a = 15 cm and b = 16 cm. In all cases an applied voltage of 20 kV was used, except a = 15 kV, b = 25 kV and c = 16 kV.
